# Prevalence of dry eye syndrome and diabetic retinopathy in type 2 diabetic patients

**DOI:** 10.1186/1471-2415-8-10

**Published:** 2008-06-02

**Authors:** Masoud Reza Manaviat, Maryam Rashidi, Mohammad Afkhami-Ardekani, Mohammad Reza Shoja

**Affiliations:** 1 Associate Professor of Ophthalmology, Yazd Diabetes Research Center, Yazd, Iran; 2 Yazd Diabetes Research Center, Yazd, Iran; 3 Associate Professor of Endocrinology, Yazd Diabetes Research Center, Yazd, Iran; 4 Professor of Ophthalmology, Yazd Diabetes Research Center, Yazd, Iran

## Abstract

**Background:**

This study was performed to assess the prevalence of dry eye syndrome and diabetic retinopathy (DR) in type 2 diabetic patients and their contributing factors.

**Methods:**

199 type 2 diabetic patients referred to Yazd Diabetes Research Center were consecutively selected. All Subjects were assessed by questionnaire about other diseases and drugs. Dry eye syndrome was assessed with Tear break up time tests and Schirmer. All the subjects underwent indirect ophthalmoscopy and retinal color photography. DR was graded according to early Treatment Diabetic Retinopathy (ETDRS) criteria.

**Results:**

Of 199 subjects, 108 patients (54.3%) suffer from dry eye syndrome. Although dry eye syndrome was more common in older and female patients, this association was not significant. But there was significantly association between dry eye syndrome and duration of diabetes (P = 0.01). Dry eye syndrome was more frequent in diabetic patients with DR (P = 0.02). DR was found in 140 patients (70.35%), which included 34 patients (17.1%) with mild non proliferative DR (NPDR), 34 patients (17.1%) with moderate NPDR, 22 patients (11.1%) with severe NPDR and 25 patients (25.1%) with proliferative DR (PDR). There were significant relation between age, sex and duration of diabetes and DR.

**Conclusion:**

In this study the prevalence of dry eye syndrome was 54.3%. Diabetes and dry eyes appear to have a common association. Further studies need to be undertaken to establish an etiologic relationship. However, examination for dry eye should be an integral part of the assessment of diabetic eye disease.

## Background

Diabetes is one of the most common leading causes of blindness in 20–74-year old persons [1]. Cataract and retinopathy are well-known as ocular complications of diabetes. Recently, problems involving the ocular surface, dry eyes in particular, have been reported in diabetic patients [1]. These patients suffer from a variety of corneal complications including superficial punctuate keratopathy, trophic ulceration, and persistent epithelial defect [2]. Dry eye is an important contributor to these problems. Dry eye syndrome has many causes. One of the most common reasons for dryness is aging process [4]. The mechanism responsible for dry eyes is unclear [5], but autonomic dysfunction may be responsible [6]. Aldose reductase, the first enzyme of the sorbitol pathway, may also be involved. The oral administration of aldose reducetase inhibitors has been shown to improve the tear dynamics significantly [7]. In one study a correlation was found between the glycated hemoglobin (HbA1C) and the presence of dry eye syndrome. The higher the HbA1c values, the higher the rate of dry eye syndrome [8]. In another study founded that diabetic patients had lower values of tear secretion and values of tear break up time test (TBUT) than control group [9]. Jin et al showed that patients with type 2 diabetes tend to develop tear film dysfunction. This study suggests that TBUT should be a routine ophthalmologic test in diabetic patients [10]. Dry eye can lead to vision deficit, scarring and perforation of the cornea and secondary bacterial infection. If this syndrome is diagnosed at first stage and treated, would be protected from its complications [2]. Therefore early diagnosis of dry eye syndrome in diabetic patients is important for beginning of treatment in early stages. Nevertheless studies to evaluate the prevalence of dry eye syndrome in type 2 diabetic patients are lacking. Therefore, we evaluated prevalence of dry eye syndrome in type 2 diabetic patients.

## Methods

Among of diabetic patients referred to Yazd Diabetes Research Center, 200 type 2 diabetic patients, including new and review cases of diabetes (was diagnosed according to ADA criteria) [1] were consecutively selected.

Clinical data of all patients which included sex, age, duration of diabetes as well as a history of other diseases, were obtained by reviewing the medical records and direct patient interview.

Subjects with secondary diabetes and those who on medication or have other diseases that can affect tear production were excluded.

Exclusion criteria included cigarette smoking, contact lens, Lasic surgery, allergies, Sjogren's syndrome, rheumatoid arthritis, Parkinson, lupus, some medications such as antihistamines, tricyclic antidepressants, oral contraceptives, and drugs used to treat high blood pressure and diuretics. Moreover vitamin A deficiency and pregnancy were excluded.

Dry eyes were suspected on the basis of a history of ocular discomfort, including soreness, gritty sensation, itchiness, redness, blurred vision that improves with blinking and excessive tearing. The condition was confirmed by ocular surface dye staining pattern with fluorescein, tear film break up time (TBUT) (value 15s) and Schirmer test (value 15 mm in 5 min), according to American Academy of Ophthalmology by a specialist [11]. Diagnosis was established by positivity one or more of the tests (TBUT or Schirmer test). Structures of the eye were assessed with slit lamp biomicroscopy examination. Retinal status was evaluated by indirect ophthalmoscopy after dilation by Tropicamid drop and retinal color photography. Diabetic retinopathy was graded according to early Treatment Diabetic Retinopathy (ETDRS) criteria [12].

Informed consent was obtained from all subjects and the research had the approval of the institutional review board and ethics committee of the Yazd University of Medical Sciences and was carried out in accordance with the Declaration of Helsinki.

### Statistical methods

Statistical analysis was performed using Statistical Package for Social Sciences (SPSS version 12.0, Chicago IL). Chi square test and t- student test was used to compare discrete variables. Significance was considered to be P < 0.05. Results were given with their 95% CIs. Data were presented as means ± SD.

## Results

In this study 200 diabetic patients were assessed. One patient was excluded from study because of trachoma.

The mean age of subjects (80 men, 119 women) was 54.16 ± 11.02 years. Of 199 subjects, 108 patients (54.3%) had dry eye syndrome which 69(58%) were female and 39(48.8%) were male. But there was not a significant association between sex and frequency of dry eye syndrome (P = 0.2) (Table 1) [See Additional file1]. Frequency of dry eye syndrome in 65–85 year old group was highest(66.7%) and in 27–41- year old group was lowest, but this correlation was not significant(P = .9) (Chart1).

Of 108 patients with dry eye syndrome the mean duration of diabetes was 11.48 ± 7.4 years whereas this was 9 ± 6.5 years in subjects without dry eye syndrome. A significant association was observed between duration of diabetes and frequency of dry eye syndrome (P = .01). Dry eye syndrome was significantly higher in subjects with DR (59.3%) (p = .02) (Table 2) [See Additional file1].

Of 108 patients with dry eye syndrome, 36.18% suffered from gritty sensation, 19.09% had soreness whereas none of them complains from pain and tearing. 11.5% had abnormal both TBUT and Schirmer's test and 29.64% of subjects had abnormal corneal staining. Corneal lesion, conjunctivitis, keratopathy and filamentry were not observed. The prevalence of DR in men was 76.2% (The retinopathy was mild in 10 patients (12.5%), whereas 13(16.3%) patients had moderate NPDR, 14(17.5%) had sever NPDR and 24(30%) had PDR.

The prevalence of DR in women was 66.4%. The retinopathy was mild in 24 patients (20.2%), whereas 21(17.6%) patients had moderate NPDR, 8(6.7%) had sever NPDR and 26(21.8%) had PDR. There was a significant association between sex and grades of DR (P = .04) (Table3) [See Additional file1]. Prevalence of DR in 55–64 year old group was highest (84.9%) and in 24–49 year old was lowest (51.5%) (Chart 2) (Table 4) [See Additional file1].

Frequency of DR was significantly higher in patients with longer duration of diabetes, it was 31.9% in patients with a history of diabetes less than 5 years, 12.48% in 5–14 years and 100% in 15 years or more (P < .0001).

## Discussion

Of 199 subjects, 108 patients (54.3%) had dry eye syndrome. Prevalence of dry eye syndrome was significantly higher in longer duration of diabetes, but sex and age did not seem to affect dry eye syndrome.

Some studies evaluated dry eye syndrome in diabetic patients. In a cohort study on 3722 subjects were aged 48 to 91 years (65 ± 10 years) and 43% male. The overall prevalence of dry eye was 14.4%. Prevalence varied from 8.4% in subjects younger than 60 years to 19.0% in those older than 80 years. Age-adjusted prevalence in men was 11.4% compared with 16.7% in women [13]. In another study a group of 140 patients aged 24–93, suffering from dry eye syndrome were assessed. A larger number of dry eye syndrome cases were identified in female patients, especially aged over 50 (80% of female and 20% of male). The most frequent general medical conditions diagnosed in the group of patients were as follows: arterial hypertension (men and women) and diabetes (women) [14]. In one study during the 5-year interval between examinations, a history of dry eye developed in 322 of 2414 subjects, for an incidence of 13.3%. Incidence was significantly associated with age. After adjusting for age, incidence was greater in subjects with a history of allergy or diabetes, who used antihistamines or diuretics, and with poorer self-rated health [15]. A cross- sectional study assessed one hundred patients with diabetes mellitus. Multiple regression analysis using the Schirmer test as a dependent variable and controlling for all the independent variables showed an association with autonomic neuropathy. No significant association was observed with the other variables, including the presence of auto antibodies. This study suggests that the low tear production seem in some DM patients is related to dysfunction of the autonomic nervous system [7]. Seifart et al compared 92 patients with diabetes types I and II and aged from 7 to 69 years with a group of normal healthy controls comparable in number, age and sex. The results show that 52.8% of all diabetic subjects complained of dry eye symptoms, as against 9.3% of the controls. They concluded close monitoring of diabetic patients and good blood sugar regulation is important for the prevention of dry eye syndrome and retinopathy [8]. In Jin study 100 patients with type II diabetes were compared with 80 normal healthy controls. In this study TBUT was significantly lower in type 2 diabetic patients [10]. In Goebels study Schirmer test and tearing reflex was significantly lower in diabetic patients compared with control group [16]. Jain reviewed the cases of 400 patients with dry eyes referred to a tertiary referral center. Of these, 80 (20%) had diabetes. Only two (2.5%) of these patients had Sjogren's syndrome, which could account for the dry ocular surface. In all the other patients, no other conditions were found to be a risk factor for dry eyes, and it was therefore presumed to be of diabetic origin [17]. In other study the tests were carried out on a 100 individuals (50 healthy subjects in control group and 50 subjects suffering from diabetes) age 50–70 years. In that group of diabetic patients (N = 50) they found that 37 (74%) of them had lower values of tear secretion. 23 (46%) of them had lower values of TBUT. In the control group (N = 50) they found that 28 (56%) had lower values of tear secretion and 17 (34%) of them had lower values of TBUT [9]. In our study frequency of dry eye syndrome was higher in diabetic patients with DR, but we did not find any study like that and larger studies need to evaluate relation between dry eye and DR.

Prevalence of dry eye in our study is very high. It might be due to aging, dry weather in this region and high prevalence of neurological disorder in type 2 diabetic patients.

Lack of control group and glycemic parameters assessment especially HbA1C could be mentioned as limitation of our study.

Of 199 diabetic patients, 140 patients (70.35%) had DR. The retinopathy was mild in 34 patients (17.1%), whereas 34(17.1%) patients had moderate NPDR, 22(11.1%) had sever NPDR and 50(25.1%) had PDR. These findings are higher than previous studies [18,12,22].

In our study prevalence of DR significantly increased with increasing of age, but it was not true in 65–82-year old subjects, and prevalence of DR in this group decreased. Some studies showed that the prevalence of DR in late-onset diabetic patients was lower than young-onset diabetic patients [19,20]. A prevalence study was undertaken to estimate the prevalence of DR in patients diagnosed as having DM after the age of 70 years. Of 150 patients examined 21(14%) had some form of DR and 10 of these patients (6.6%) had threaten DR. Those patients with DR had a significantly higher median duration of diabetes (5.0 years) compared with those patients without DR (3.5 years) [21].

In our study there was significant association between sex and grades of DR. Lower grades of DR was more common in women and higher grades of DR was more common in men, such a relation was found in Rema et al [12]

Our results showed a significant association between prevalence of DR and diabetes duration, this pattern was seen in Klein et al. As the prevalence of DR varied from 28.8% in persons who had diabetes for less than five years to 77.8% in persons who had diabetes for 15 or more years [22].

## Conclusion

Our findings support the impression that diabetic patients have an elevated prevalence of dry eye syndrome. Diabetic retinopathy and dry eye appear to have a common association. Further studies need to be undertaken to establish an etiologic relationship. However, examination for dry eyes should be an integral part of the assessment of diabetic eye disease.

## Competing interests

The authors declare that they have no competing interests.

## Authors' contributions

MRM participated in the design of the study and coordination and carried out ophthalmologic examination and revising the manuscript. MR helped with study arrangement, interpretation of data and drafted the manuscript. MA-A participated in the design of the study and coordination. MRS participated in the design of the study. All authors read and approved the final manuscript.

## Pre-publication history

The pre-publication history for this paper can be accessed here:


